# Chronic pain in the elderly: Exploring cellular and molecular mechanisms and therapeutic perspectives

**DOI:** 10.3389/fragi.2024.1477017

**Published:** 2024-09-12

**Authors:** Mario García-Domínguez

**Affiliations:** ^1^ Program of Immunology and Immunotherapy, CIMA-Universidad de Navarra, Pamplona, Spain; ^2^ Department of Immunology and Immunotherapy, Clínica Universidad de Navarra, Pamplona, Spain; ^3^ Centro de Investigación Biomédica en Red de Cáncer (CIBERONC), Madrid, Spain

**Keywords:** nociceptor, chronic pain, pro-inflammatory cytokine, peripheral and central sensitization, neuroplasticity, analgesic effects

## Abstract

Chronic pain is a debilitating condition frequently observed in the elderly, involving numerous pathological mechanisms within the nervous system. Diminished local blood flow, nerve degeneration, variations in fiber composition, alterations in ion channels and receptors, accompanied by the sustained activation of immune cells and release of pro-inflammatory cytokines, lead to overactivation of the peripheral nervous system. In the central nervous system, chronic pain is strongly associated with the activation of glial cells, which results in central sensitization and increased pain perception. Moreover, age-related alterations in neural plasticity and disruptions in pain inhibitory pathways can exacerbate chronic pain in older adults. Finally, the environmental influences on the development of chronic pain in the elderly must be considered. An understanding of these mechanisms is essential for developing novel treatments for chronic pain, which can significantly improve the quality of life for this vulnerable population.

## 1 Introduction

The International Association for the Study of Pain (IASP) defines *pain* as an “unpleasant sensory and emotional experience associated with, or resembling that associated with, actual or potential tissue damage” (Raja et al., 2020). Pain is initiated by nociceptors, specialized neurons located in the sensory ganglia of the peripheral nervous system (PNS). These neurons subsequently translate different stimuli into an action potential and transmit electrical impulses to the spinal cord and brainstem, reaching some areas of the brain, where pain is experienced (Gore, 2022).

Pain can be classified into various types based on distinct characteristics. *Acute pain* typically originates from an injury and is distinguished by its temporary period (Bonezzi et al., 2020). *Chronic pain* persists for a prolonged duration, and frequently extends beyond the timeframe required for tissue healing (Bonezzi et al., 2020). Its underlying causes are different, such as tissue injury, inflammation, and chemotherapy (Bonezzi et al., 2020). Moreover, chronic pain usually coexists with other medical conditions, like depression, and sleep disturbances (Dahan et al., 2014). Risk factors that contribute to the development of chronic pain include age, gender, tobacco and alcohol consumption, weight, and mental health (Mills et al., 2019). Survey-based studies in Europe show that chronic pain prevalence increases with age, estimated between 38% and 60% in individuals aged over 65 (Larsson et al., 2016). Older adults are particularly vulnerable due to increased prevalence of some painful diseases, which contribute to a deterioration in their overall health (Lam et al., 2024). Managing chronic pain requires a deep understanding of its underlying causes and the adoption of patient-centered therapies (Mayoral Rojals et al., 2022).

This review will begin with an examination of the anatomy and physiology of pain transmission. Subsequently, the cellular and molecular mechanisms that contribute to high prevalence of chronic pain among the older adults will be analyzed. Finally, the management strategies for chronic pain in the elderly will be examined.

## 2 Anatomy and physiology of pain

**FIGURE 1:**
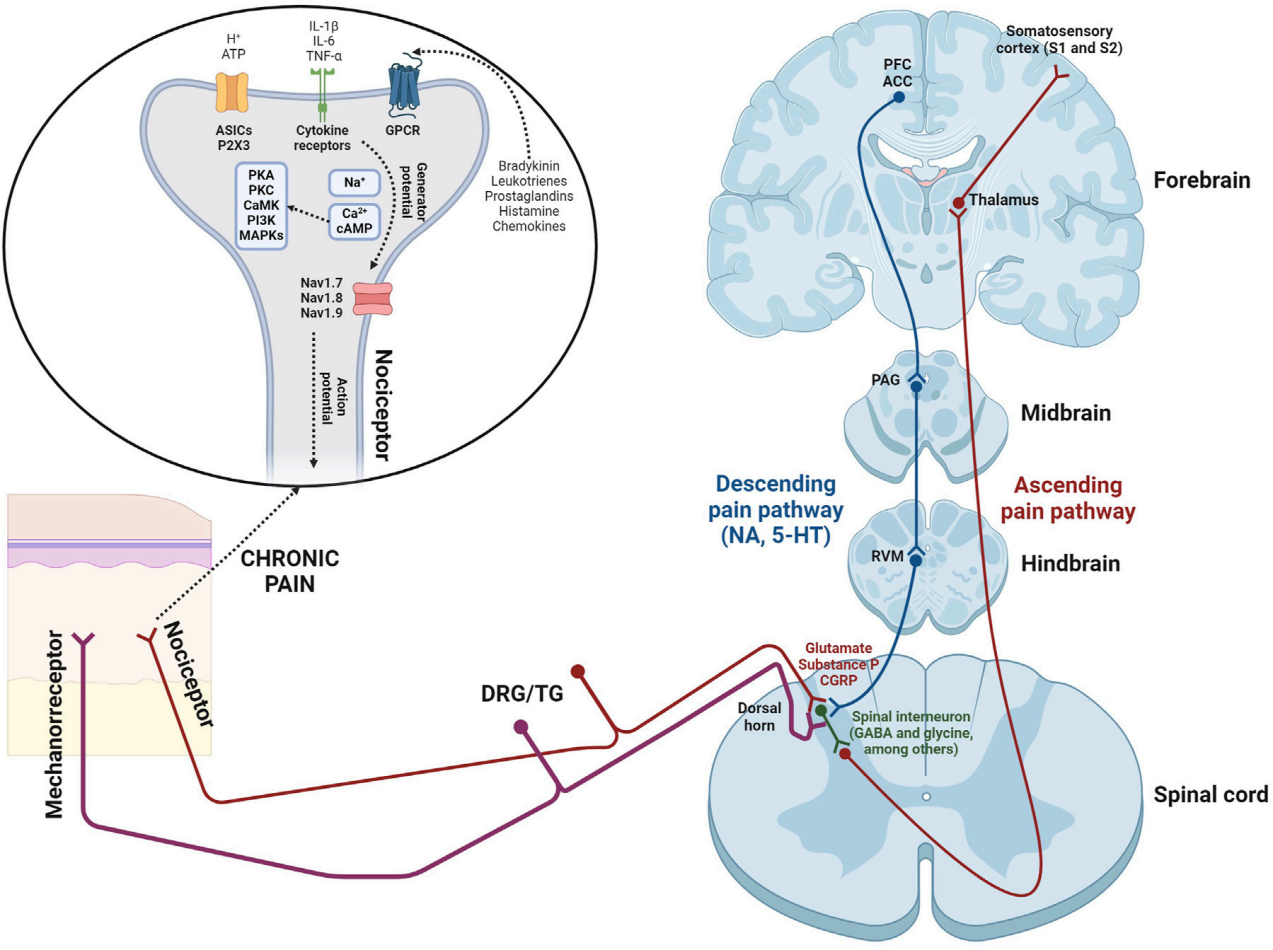
Scheme illustrating the pain transmission, showing both ascending and descending pathways. Additionally, the influence of non-noxious sensory afferents (mechanoreceptors) is also represented, which induces an analgesic effect, according to the gate control theory proposed by Ronald Melzack and Patrick Wall in 1965 (Melzack and Wall, 1965). Abbreviations: IL-1β and IL-6 (interleukin-1 beta and interleukin-6); TNF-α (tumor necrosis factor-alpha); ATP (adenosine triphosphate); ASICs (acid sensing ion channels); P2X3 (P2X purinergic receptor type 3); GPCR (G protein-coupled receptors); PKA (protein kinase A); PKC (protein kinase C); CaMK (Ca^2+^/calmodulin-dependent protein kinase); PI3K (phosphatidylinositol 3-kinase); MAPKs (mitogen-activated protein kinases); cAMP (cyclic adenosine monophosphate); Nav1.7, 1.8, and 1.9 (sodium voltage-gated channel alpha subunits 9, 10, and 11); DRG/TG (dorsal root ganglia/trigeminal ganglia); GABA (γ-aminobutyric acid); CGRP (calcitonin gene-related peptide); NA (noradrenaline); 5-HT (serotonine); RVM (rostral ventromedial medulla); PAG (periaqueductal grey); PFC (prefrontal cortex); ACC (anterior cingulate cortex).

Pain is recognized as a protective mechanism that identifies tissue damage. However, it can also become pathological, causing physical and psychosocial issues when the problem persists beyond the initial injury (Abdel Shaheed et al., 2024). To understand effectively both the anatomy and physiology of pain, it’s essential to explore numerous processes by which a peripheral painful stimulus reaches the brain. This pathway requires four pivotal steps: (i) transduction; (ii) transmission; (iii) modulation; (iv) perception (Yam et al., 2018). Key elements in pain transmission will be detailed below (Figure 1).

1. Nociceptors. The transformation of a painful stimulus into an action potential occurs within free nerve endings known as *nociceptors.* Nociceptors, which originate from cell bodies in the dorsal root ganglia (DRG) and/or trigeminal ganglia (TG; Dubin and Patapoutian, 2010), are activated by thermal, mechanical, and chemical stimuli (e.g., adenosine triphosphate -ATP-, leukotrienes, hydrogen ions -H^+^-, prostaglandins, histamine, bradykinin, cytokines, and chemokines; Basbaum et al., 2009). Nociceptor activation begins when receptors detect stimuli, leading to production of generator potentials. This mechanism results in the influx of Na^+^ and Ca^2+^ ions, and subsequent membrane depolarization. If the depolarization reaches a certain threshold, voltage-gated sodium channels (Nav1.7, Nav1.8, and Nav1.9) are strongly activated. This results in rapid membrane depolarization and the generation of an action potential (Basbaum et al., 2009).
2. Spinal cord. Nociceptive afferents terminate in the spinal dorsal horn, where they form synapses with second-order neurons through the secretion of glutamate, substance P, and calcitonin gene-related peptide (CGRP; Basbaum et al., 2009). Ascending tracts, which transmit noxious stimuli, project through the ventrolateral spinal cord to reach some nuclei of the thalamus (Gore, 2022).
3. From thalamus to brain (ascending tract). The thalamus plays a fundamental role in processing somatosensory information, including pain signals. Third-order neurons, emanating from thalamic nuclei, project to the somatosensory cortex (primary -S1- and secondary -S2-), anterior cingulate cortex (ACC), prefrontal cortex (PFC), periaqueductal grey (PAG), nucleus accumbens (NAc), and hippocampus (HP; Gore, 2022).
4. From brain to spinal cord (descending tract). The descending pain pathway is composed of some supraspinal elements, including the rostral ventromedial medulla (RVM) and PAG (Bourne et al., 2014). Noradrenaline (NA) and serotonin (5-HT) are the key neurotransmitters involved in the descending inhibition (Bourne et al., 2014). The PAG receives inputs from the prefrontal cortex and ACC, and activates some neurons located in the nucleus raphe magnus (NRM), which contain 5-HT. Serotoninergic neurons, along with noradrenergic projections (originating from the locus coeruleus -LC-), establish synapses with spinal interneurons (Ossipov et al., 2014). These interneurons, located in the spinal dorsal horn, release several inhibitory neurotransmitters such as glycine, γ-aminobutyric acid (GABA), nitric oxide (NO), opioid peptides, and endocannabinoids. In accordance with the *gate control theory*, proposed by Ronald Melzack and Patrick Wall in 1965 (Melzack and Wall, 1965), non-noxious sensory afferents (mechanorreceptors) activate spinal interneurons, thereby modulating pain signals within the spinal cord. The analgesic effects mediated by inhibitory neurotransmitters are as follows: (i) activation of the respective receptors, located on nociceptive afferents, induces the influx of Cl⁻ ions or efflux of K⁺ ions, leading to hyperpolarization and a subsequent reduction in the secretion of glutamate, substance P, or CGRP onto the spinal dorsal horn; (ii) the inhibition of second-order neurons due to their hyperpolarization, a process that occurs in a similar manner (Hughes and Todd, 2020).

## 3 Peripheral mechanisms of chronic pain in the elderly

**FIGURE 2:**
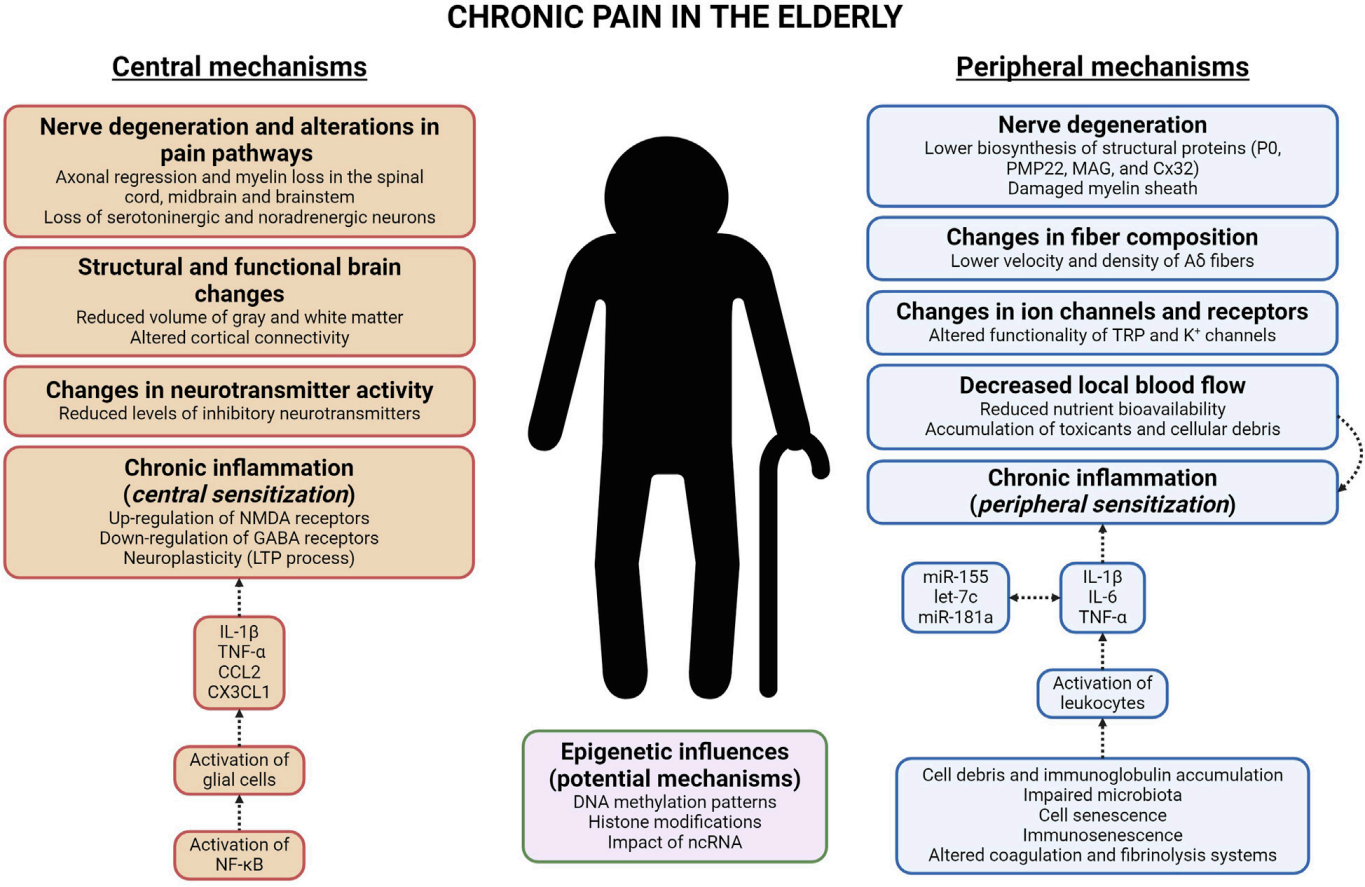
Diagram that represents the cellular and molecular mechanisms, both peripheral and central, which induce chronic pain in the elderly. Abbreviations: NMDA (N-methyl-D-aspartate); GABA (γ-aminobutyric acid); LTP (long-term potentiation); IL-1β and IL-6 (interleukin-1 beta and interleukin-6); TNF-α (tumor necrosis factor-alpha); CCL2 and CX3CL1 (chemokine -C-C motif-ligand two and chemokine -C-X3-C motif-ligand 1); NF-κB (nuclear factor-κB); ncRNA (non-coding RNA); P0 (myelin protein zero); PMP22 (peripheral myelin protein 22); MAG (myelin-associated glycoprotein); Cx32 (connexin-32); TRP (transient receptor potential).

The aging process induces numerous changes in the PNS, which may play a fundamental role in the generation and maintenance of chronic pain. A deep understanding of these mechanisms is crucial for developing innovative therapies that improve the quality of life for older adults with chronic pain. The mechanisms which contribute to this circumstance are the following (Figure 2).

1. Nerve degeneration. Is the major contributor to chronic pain in the older adults. One significant alteration is the degeneration of the *myelin sheath*, the protective barrier surrounding nerve fibers, which slows nerve conduction and impairs signal transmission (Kemp et al., 2014). This fact has been associated with a decrease in the biosynthesis of numerous structural proteins (P0, PMP22, MAG, and Cx32), crucial for maintaining the integrity of the myelin sheath (Verdú et al., 2000).
2. Changes in fiber composition. In the elderly, there is a profound reduction in both the conduction velocity and density of specific Aδ nociceptive nerve fibers (Chakour et al., 1996). This alteration in fiber composition results in chronic pain (Gibson and Farrell, 2004).
3. Changes in ion channels and receptors. Aging causes significant modifications in nociceptor ion channels, affecting both their quantity and type, which contributes to the increased prevalence of chronic pain in the elderly. Moreover, aging alters the functionality of transient receptor potential channels (TRP; e.g., TRPV1) as well as K^+^ channels (e.g., K_Ca_2.1), causing increased nociceptor activity and release of substance P and CGRP onto the spinal dorsal horn, thereby intensifying pain sensation (Strickland et al., 2019; Xiao et al., 2023).
4. Decreased local blood flow. Recent research, conducted by Devanne et al. (2024), has revealed that diminished local blood flow in older adults induces chronic inflammation and amplified pain sensitivity. This situation arises from reduced nutrient bioavailability, as well as the accumulation of toxicants (like reactive oxygen species -ROS-) and cell debris (Sanada et al., 2018; Chaudhary et al., 2023).
5. Chronic inflammation. In the elderly, chronic inflammation is usually observed in many tissues. Pro-inflammatory cytokines (e.g., IL-1β, IL-6, and TNF-α), regulated by many microRNAs (e.g., miR-155, let-7c, and miR-181a), are known to disrupt many anabolic signaling pathways, which can lead to development of sarcopenia (Fan et al., 2016). The factors which contribute to the chronic inflammation are as follows (Sanada et al., 2018): (i) cell debris and/or immunoglobulin accumulation (this fact results in the sustained activation of the immune system); (ii) impaired microbiota (evokes the infiltration of microorganisms and the stimulation of the immune system); (iii) cell senescence (*senescent cells* -generated by several mechanisms, like telomere shortening, which culminate in the activation of p16 and p53-release several pro-inflammatory cytokines, resulting in low-grade inflammation); (iv) immunosenescence (age-related reduction in immune responses; Liu et al., 2023); (v) altered coagulation and fibrinolysis systems (recent studies have observed an increase in inflammatory processes among older adults). Chronic inflammation in the periphery evokes *peripheral sensitization*, defined by elevated responsiveness of nociceptors (Puja et al., 2021).
6. Epigenetic influences. Research on biological aging identifies nine potential hallmarks, including epigenetic alterations (López-Otín et al., 2013). These variations are distinguished by anomalous DNA methylation patterns, histone modifications, and the participation of ncRNAs, particularly microRNAs (Wang et al., 2022). Although the specific genes influenced by epigenetic alterations in the PNS of older adults with chronic pain are not yet identified, genes influenced by epigenetic mechanisms have been identified in several experimental chronic pain murine models, making it a promising research area. In the spinal nerve ligation (SNL) experimental model, which induces chronic neuropathic pain, the activation of histone methyltransferase G9a (dimethylates histone H3 at lysine 9; H3K9me2) causes transcriptional repression of the *Oprm1* gene (encodes μ-opioid receptor -MOR-) in the DRG (Zhang et al., 2016). Moreover, this model alters DNA methylation patterns in the DRG, influencing genes that encode voltage-gated and ligand-gated ion channels, all of which are involved in pain processing (Garriga et al., 2018). The role of microRNAs should also be considered, due to the detection of 63 microRNAs whose level of expression was altered in the DRG following SNL (von Schack et al., 2011).

## 4 Central mechanisms of chronic pain in the elderly

Chronic pain in the elderly is influenced by complex changes in the central nervous system (CNS) that result in altered pain perception. These effects are linked to structural and functional alterations in brain regions involved in pain processing. Consistent with the peripheral mechanisms of chronic pain, understanding central mechanisms is crucial for developing novel pain management strategies tailored to older adults. The mechanisms which contribute to this situation are as follows (Figure 2).

1. Nerve degeneration and alterations in pain pathways. In older adults, there are many degenerative changes in the spinal dorsal horn (Piekarz et al., 2020), midbrain, and brainstem (van der Meulen et al., 2024), involving axonal regression and myelin loss. Moreover, in older adults with chronic pain, there is a significant reduction in the activity of the descending pain pathway. This is linked to a progressive loss of serotonergic and noradrenergic neurons in the spinal dorsal horn (Iwata et al., 2002). This impairment leads to increased sensitivity to pain (van der Meulen et al., 2024).
2. Structural and functional brain changes. Significant structural alterations in the brain have been documented in older adults with chronic pain. These variations mainly affect regions involved in pain processing, suggesting a complex interaction between aging and pain (Mackey and Maeda, 2004). Gray matter volume reductions have been reported in many areas of the brain in the elderly (such as, PFC, PAG, HP, and corpus callosum; Zimmerman et al., 2009; Buckalew et al., 2010; Luo et al., 2022). Moreover, several studies show that older adults with chronic pain have altered cortical connectivity within the descending pain pathway (Cruz-Almeida and Cole, 2020).
3. Changes in neurotransmitter activity. Multiple inhibitory neurotransmitters (GABA, 5-HT, NA, glycine, and opioid peptides) are diminished in brains of older patients with chronic pain (Bruehl et al., 2012; Yang and Chang, 2019). The absence of these neurotransmitters in the CNS allows nociceptors to release increased amounts of glutamate and/or substance P onto the spinal dorsal horn, due to the lack of presynaptic inhibition mechanisms (Hughes and Todd, 2020).
4. Chronic inflammation. Persistent inflammation in the CNS is fundamental in the maintenance of chronic pain in the elderly. This process is characterized by the activation of glial cells, especially microglia and astroglia, in both the spinal cord and brain (Paladini et al., 2015). Glial cells secrete numerous pro-inflammatory cytokines (e.g., IL-1β and TNF-α) and chemokines (e.g., CCL2 and CX3CL1), which lead to *central sensitization* by increasing neuronal excitability and synaptic plasticity (Puja et al., 2021). This mechanism is driven by the NF-κB transcription factor (Shih et al., 2015). The release of pro-inflammatory mediators enhances pain intensity through various mechanisms: (i) upregulation of pain-related receptors (e.g., IL-1β increases the activity of N-methyl-D-aspartate -NMDA-receptors; Viviani et al., 2003); (ii) downregulation of GABA receptors (induced by TNF-α; Stellwagen et al., 2005); (iii) *neuroplasticity* (numerous cytokines trigger the long-term potentiation -LTP- process and allow continuous pain signal transmission; Gonçalves Dos Santos et al., 2020).
5. Epigenetic influences. Consistent with the previous section, the epigenetic mechanisms involved in chronic pain in the CNS of the elderly must be elucidated. However, the impact of epigenetics on chronic pain is recognized, providing a starting point for studying chronic pain in the elderly. DNA methylation has a considerable effect on chronic pain in the CNS, as evidenced by studies in rodent models of chronic neuropathic pain that show modifications in global DNA methylation within the PFC and thalamus. These alterations are associated with decreased expression of DNA methyltransferases DNMT1 and DNMT3a, along with changes in TET enzyme levels (Alvarado et al., 2015; Rodrigues et al., 2023). In relation to histone modifications, trimethylation of histone H3 (H3K27me3) and increased activity of histone deacetylases (HDACs) contribute to sustained production of pro-inflammatory cytokines in the CNS of rodents with chronic inflammatory pain (Jiang et al., 2023). In many experimental models of chronic pain, various microRNAs (including miR-101, miR-132, miR-155, and miR-223) have been identified as modulators of chronic pain through their effects on neuronal excitability and/or synaptic plasticity (Kovanur Sampath et al., 2023; Zhang et al., 2023).

## 5 Management of chronic pain in the elderly

It’s evident that chronic pain significantly affects the quality of life in the elderly. In light of this, ongoing research is uncovering new molecular mechanisms involved in chronic pain (Simonetti and Mauceri, 2023). Meanwhile, a wide range of preclinical studies and clinical trials are being conducted with innovative therapies, leading to highly promising outcomes (Chen et al., 2021; Ashar et al., 2022). Regardless, managing chronic pain in this cohort requires a multifaceted strategy that combines several pharmacological, physical, and psychological therapies (Schwan et al., 2019).

### 5.1 Pharmacological therapies

The American Geriatrics Society (AGS) provides the most appropriate treatment guidelines for managing chronic pain in the elderly, first established in 1998 and updated with newer pharmacological approaches in 2009 (American Geriatrics Society Panel on Pharmacological Management of Persistent Pain in Older Persons, 2009).

1. Nonsteroidal anti-inflamatory drugs (NSAIDs). NSAIDs are anti-inflammatory medications that inhibit prostaglandin synthesis through the cyclooxygenase (COX) pathway (Wongrakpanich et al., 2018). Classic NSAIDs (e.g., ibuprofen) are non-selective and inhibit both COX-1 and COX-2 enzymes, but recent NSAIDs (e.g., celecoxib) specifically target COX-2 (Wongrakpanich et al., 2018).
2. Anti-depressants. Anti-depressants used in the treatment of chronic pain include tricyclic anti-depressants (TCAs; e.g., imipramine) and serotonin/noradrenaline reuptake inhibitors (SNRIs; duloxetine; McCleane, 2008). Both types of drugs block the reuptake of 5-HT and NA, resulting in increased levels of these neurotransmitters in the synaptic cleft (McCleane, 2008).
3. Anti-convulsivants. Classical anti-convulsivants (such as carbamazepine) serve as inhibitors of sodium channels and reduce nerve hyperexcitability (Sullivan and Robinson, 2006). Moreover, gabapentinoids (e.g., gabapentin), which are α-2δ calcium channel blockers, influence primary afferent excitability (Sidhu and Sadhotra, 2016).
4. Other analgesics: (i) cannabinoids (like tetrahydrocannabinol -THC-), that bind to CB1 and CB2 cannabinoid receptors, have demonstrated efficacy in many clinical trials for the management of chronic pain (Johal et al., 2020); (ii) opioids (such as morphine; their effects are mediated through opioid receptors found in the midbrain and spinal cord; Guerriero, 2017); (iii) muscle relaxants (include baclofen; these drugs are linked to side effects including sedation and muscle weakness; Fu and Perloff, 2022); (iv) low-dose naltrexone (naltrexone, a non-selective opioid antagonist, exerts anti-inflammatory effects by inhibiting Toll-like receptor 4 -TLR4-, present in microglial cells; McKenzie-Brown et al., 2023); (v) memantine (a NMDA receptor antagonist that reduces pain intensity; Kurian et al., 2019).

### 5.2 Physical and psychological interventions

This section will characterize the physical and psychological interventions that complement the pharmacological treatments.

1. Physical interventions. The primary aim of rehabilitation is to improve impairment, typically through modalities that target the underlying pathophysiological causes. When improvement of impairment is unlikely, rehabilitation should instead focus on limiting patient disability (Leung et al., 2024). Examples of physical therapies include Tai Chi and high-/low-intensity strengthening programs (Leung et al., 2024).
2. Psychological interventions. Psychological treatments for older adults with chronic pain aim to manage emotional and cognitive aspects to enhance overall quality of life (Driscoll et al., 2021). Examples of psychological therapies include cognitive-behavioral therapy (CBT) and mindfulness-based stress reduction (MBSR) techniques (Driscoll et al., 2021).

## 6 Conclusion

The analysis of chronic pain in the elderly reveals that aging-related changes in neuroplasticity, chronic low-grade inflammation, and dysfunction of pain inhibitory pathways. Additionally, epigenetic modifications, along with PNS degeneration and diminished local blood flow, increase pain sensitivity. These insights highlight the complexity of chronic pain in the elderly and underscore the necessity for targeted therapeutic interventions to manage their pain effectively.
